# Streptococcus pyogenes erythromycin resistance in Italy.

**DOI:** 10.3201/eid0502.990223

**Published:** 1999

**Authors:** M Bassetti, E Mantero, G Gatti, A Di Biagio, D Bassetti


importance of this symptomatic shedding in transmission among preschool children is well established (3); however, that of symptom-free shedding in adults is unknown. We report here that the rate of symptom-free STEC O157:H7 shedding is higher in adults 30 to 49 years of age than in others.

STEC infections have been notifiable in Japan since August 1996. When STEC is found in the feces of patients in schools, families, and hospitals, local health centers and public health institutes must test (generally using MacConkey sorbitol agar with cefixime-potassium tellurite medium) for the pathogen in stool specimens of contacts of the patients. The pathogen is also sought twice a month in the stool specimens of food-handlers. All isolates from culture-positive patients are collected by Japan's National Institute of Infectious Diseases.

In 1997, 1,412 STEC O157:H7 human isolates were examined for subtyping of Shiga toxin genes *stx1* and *stx2* by polymerase chain reaction, for genotyping by *Xba*I-digested pulsed-field gel electrophoresis (PFGE) (4,5), and for their relationship with symptoms; 1,381 isolates (from culture-positive persons with well-characterized clinical status) were further analyzed. The rates by age group among STEC O157:H7-shedding persons reporting one or more symptoms (vs. culture-positive persons without symptoms) were as follows: 82% (475 of 576) younger than 10 years old; 81% (145 of 178), 10 to 19 years; 63% (98 of 156), 20 to 29 years; 25% (32 of 128), 30 to 39 years; 34% (34 of 100), 40 to 49 years; 54% (57 of 106), 50 to 59 years; 56% (38 of 68), 60 to 69 years; 68% (47 of 69), older than 70 years. Culture-positive persons under 20 years of age, especially children under 10 years of age, were more likely to have symptoms than other age groups. Intermediate rates of symptom-free persons with positive stool cultures occurred in young adults (20 to 29 years of age) and the elderly (70 years of age), while the highest rates of stool-positive, symptom-free persons were adults, especially those between 30 and 49 years of age. In terms of pathogen virulence, we did not find significant differences in the distribution of *stx* subtypes and PFGE genotypes between strains shed by symptomatic and asymptomatic persons. These results suggest that the rate of symptom-free STEC O157:H7 shedding may be associated with age rather than organism-related factors. Possible age-related host factors that could influence the presence of STEC O157:H7 in the stools of symptom-free persons include qualitative and quantitative differences in intestinal cross-reactive antibodies against STEC O157:H7, intestinal bacterial flora, or the sensitivity to Stx toxins between children and adults. Further investigations will be required to determine the relative importance of these and other host factors. Our finding of a high rate of asymptomatic shedding in adults may suggest the potential for secondary transmission of the bacteria from symptom-free STEC O157:H7-shedding adults to healthy children.

**Acknowledgments**

We thank investigators of prefectural and municipal public health institutes for their collaboration.

This work was supported by a grant from the Ministry of Health and Welfare of Japan.

**Jun Terajima, Hidemasa Izumiya, Akihito Wada, Kazumichi Tamura, and Haruo Watanabe**
National Institute of Infectious Diseases, Tokyo, Japan

### References

1. Tarr PI. *Escherichia coli* O157:H7: clinical, diagnostic, and epidemiological aspects of human infection. Clin Infect Dis 1995;20:1-8.
2. Belongia EA, Osterholm MT, Soler JT, Ammend DA, Braun JE, MacDonald KL. Transmission of *Escherichia coli* O157:H7 infection in Minnesota child day-care facilities. JAMA 1993;269:883-8.
3. Karch H, Russman H, Schmidt H, Schwarzkopf A, Heesmann J. Long-term shedding and clonal turnover of enterohemorrhagic *Escherichia coli* O157:H7 in diarrheal diseases. J Clin Microbiol 1995;33:1602-5.
4. Watanabe H, Wada A, Inagaki Y, Itoh K, Tamura K. Outbreaks of enterohaemorrhagic *Escherichia coli* O157:H7 infection by two different genotype strains in Japan. Lancet 1996;348:831-2.
5. Izumiya H, Terajima J, Wada A, Inagaki Y, Itoh K, Tamura K, et al. Molecular typing of enterohemorrhagic *Escherichia coli* O157:H7 isolates in Japan by using pulsed-field gel electrophoresis. J Clin Microbiol 1997;35:1675-80.

### *Streptococcus pyogenes* Erythromycin Resistance in Italy

**To the Editor:** *Streptococcus pyogenes* resistance to erythromycin began to emerge as a serious problem worldwide in the early 1990s. In some areas in Italy, 30% to 40% of strains have beome resistant (1-3). Throughout Italy, the use of macrolides, particularly the newest ones (azithromycin and clarithromycin), has increased in the treatment of infections caused by Group A streptococci. This therapeutic approach is contrary to current guidelines, which recommend using betalactam antibiotics as first-choice therapy and reserving macrolides only for patients allergic to betalactams.

In 1997 in Finland, a decrease was observed in the use of macrolide antibiotics in ambulatory patients from 2.40 defined daily doses per 1,000 inhabitants in 1991 to 1.38 in 1992. Subsequently, the maintenance of doses at 1.28 to 1.74 defined daily doses resulted in a substantial decrease in the percentage of group A streptococcal resistance to erythromycin, reported as 16.5% in 1992, 19% in 1993, 15.6% in 1994, 10% in 1995, and 8.6% in 1996 (4). These data prompted us to evaluate such phenomena in our geographic area, the urban area of Genoa, Italy (approximately 120,000 inhabitants).

From January 1991 to June 1998, 311 (6.1%) of 5,117 strains of *S. pyogenes* throat swabs from patients with pharyngotonsillitis were isolated. We observed a higher number of group A streptococci isolates from throat swabs starting in 1996 than we had in 1991 to 1995 (chi-square = 35.653, $p < 0.0001$). All isolates were tested for susceptibility to penicillin and erythromycin by standard susceptibility tests (broth microdilution) as recommended by the National Committee for Clinical Laboratory Standards. All isolates were susceptible to penicillin. From 1991 to 1996, the percentage of *S. pyogenes* resistant or with intermediate resistance to erythromycin increased from 0% to 50% (1992, 6%; 1993, 13%; 1994, 14%; 1995, 24%; 1996, 50%). In 1997 and the first half of 1998, resistance to erythromycin decreased to 39% and 34%, respectively. The number of resistant strains before 1996 was significantly lower than from 1996 to 1998 (chi-square = 50.386, $p < 0.0001$). Analysis of antibiotic consumption in our district showed an increase in the use of macrolides (erythromycin and the new compounds clarithromycin and azithromycin) from 0.445 defined daily dose per 1,000 inhabitants in 1994 to 1.140 in 1996. In 1997 and in the first half of 1998, consumption decreased to 0.9 and 0.8, respectively; we observed a correlation between the number of resistant isolates and the defined daily dose increase (correlation [$R^2$] = 0.795, $p = 0.0153$).

*S. pyogenes* resistance to erythromycin rose from 6% to 50% in only 4 years and then rapidly decreased from 50% to 34% in an 18-month period, corresponding to a 57% decrease in defined daily dose (from 1.41 in 1996 to 0.8 in the first half of 1998). Our data suggest that *S. pyogenes* resistance to erythromycin is associated with frequency of macrolide use.

**Matteo Bassetti, Enrico Mantero, Giorgio Gatti, Antonio Di Biagio, and Dante Bassetti**
University of Genoa, G. Gaslini Children's Hospital, Genoa, Italy

### References

1. Cipriani P, Debbia EA, Gesu GP, Menozzi MG, Nani E, Nicolosi V, et al. Indagine policentrica AMCLI sull'incidenza di resistenze agli antibiotici in *S. pyogenes*. Microbiologia Medica 1995;10:171-4.
2. Cornaglia G, Ligozzi M, Mazzariol A, Valentini M, Orefici G, Fontana R. Rapid increase of resistance to erythromycin and clindamycin in *Streptococcus pyogenes* in Italy, 1993-1995. Emerg Infect Dis 1996;2:339-42.
3. Cocuzza C, Blandino G, Mattina R, Nicoletti F, Nicoletti G. Antibiotic susceptibility of group A streptococci in 2 Italian cities: Milano and Catania. Microb Drug Resist 1997;3:379-84.
4. Seppala H, Klaukka T, Vuopio-Varkila J, Muotiala A, Helenius H, Lager K, et al. The effect of changes in the consumption of macrolide antibiotics on erythromycin resistance in group A streptococci in Finland. Finnish Study Group for Antimicrobial Resistance. N Engl J Med 1997;337:441-6.

### Estimated Incidence of *Clostridium difficile* Infection

**To the Editor:** Since the publication of our article Increasing hospitalization and death, possibly due to *Clostridium difficile* diarrheal disease (1), we have received several requests to estimate the incidence of *C. difficile* infection. Our original study included only hospitalized patients treated at the Lovelace Medical Center from 1993 to 1996, and no information on the incidence of *C. difficile* infection. In response to these requests, we used inpatient and outpatient medical claims for the Lovelace managed care population to calculate incidence rates. We searched medical claims for the Lovelace Health Plan/Senior Plan (LHP) to identify patients who had a *C. difficile* diagnosis between January 1, 1993, and December 31, 1997. LHP members are residents of New Mexico, most residing in or near Albuquerque.

